# Non-invasive Assessment of Systolic and Diastolic Cardiac Function During Rest and Stress Conditions Using an Integrated Image-Modeling Approach

**DOI:** 10.3389/fphys.2018.01515

**Published:** 2018-10-30

**Authors:** Belén Casas, Federica Viola, Gunnar Cedersund, Ann F. Bolger, Matts Karlsson, Carl-Johan Carlhäll, Tino Ebbers

**Affiliations:** ^1^Division of Cardiovascular Medicine, Department of Medical and Health Sciences, Linköping University, Linköping, Sweden; ^2^Center for Medical Image Science and Visualization (CMIV), Linköping University, Linköping, Sweden; ^3^Department of Biomedical Engineering, Linköping University, Linköping, Sweden; ^4^Department of Medicine, University of California, San Francisco, San Francisco, CA, United States; ^5^Division of Applied Thermodynamics and Fluid Mechanics, Department of Management and Engineering, Linköping University, Linköping, Sweden; ^6^Department of Clinical Physiology, Department of Medical and Health Sciences, Linköping University, Linköping, Sweden

**Keywords:** computational modeling, phase-contrast magnetic resonance imaging, left ventricle, systolic function, diastolic function, dobutamine

## Abstract

**Background:** The possibility of non-invasively assessing load-independent parameters characterizing cardiac function is of high clinical value. Typically, these parameters are assessed during resting conditions. However, for diagnostic purposes, the parameter behavior across a physiologically relevant range of heart rate and loads is more relevant than the isolated measurements performed at rest. This study sought to evaluate changes in non-invasive estimations of load-independent parameters of left-ventricular contraction and relaxation patterns at rest and during dobutamine stress.

**Methods:** We applied a previously developed approach that combines non-invasive measurements with a physiologically-based, reduced-order model of the cardiovascular system to provide subject-specific estimates of parameters characterizing left ventricular function. In this model, the contractile state of the heart at each time point along the cardiac cycle is modeled using a time-varying elastance curve. Non-invasive data, including four-dimensional magnetic resonance imaging (4D Flow MRI) measurements, were acquired in nine subjects without a known heart disease at rest and during dobutamine stress. For each of the study subjects, we constructed two personalized models corresponding to the resting and the stress state.

**Results:** Applying the modeling framework, we identified significant increases in the left ventricular contraction rate constant [from 1.5 ± 0.3 to 2 ± 0.5 (*p* = 0.038)] and relaxation constant [from 37.2 ± 6.9 to 46.1 ± 12 (*p* = 0.028)]. In addition, we found a significant decrease in the elastance diastolic time constant from 0.4 ± 0.04 s to 0.3 ± 0.03 s (*p* = 0.008).

**Conclusions:** The integrated image-modeling approach allows the assessment of cardiovascular function given as model-based parameters. The agreement between the estimated parameter values and previously reported effects of dobutamine demonstrates the potential of the approach to assess advanced metrics of pathophysiology that are otherwise difficult to obtain non-invasively in clinical practice.

## Introduction

Quantification of left ventricular (LV) contractility is critical to the detection and management of many cardiovascular diseases, including ischemic heart disease, valvular disorders, and heart failure. The gold standard for deriving load-independent parameters characterizing LV contractility and diastolic relaxation relies on specialized and rarely performed direct invasive measurements of LV pressures (Zile et al., 2004; Westermann et al., 2008). Among measurements that can be made from those data, the slope of the end-systolic pressure-volume relationship (ESPVR), defined as the maximal elastance of the ventricle, is a proven metric of systolic contractility (Muthurangu and Razavi, 2005). Similarly, the end-diastolic pressure volume relationship (EDPVR) is a measure of the ventricular compliance during diastole (Burkhoff et al., 2005). Extrapolation of these relationships to the entire cardiac cycle leads to the concept of time-varying elastance (Suga et al., 1973), defined as the ratio of instantaneous ventricular pressure to volume. The time-varying elastance can be used to characterize the contractile state of the ventricle at each time point during the cardiac cycle.

The clinical applicability of pressure-volume relationships has been hindered by the difficulty of obtaining these measurements non-invasively. Different imaging methods have been used to provide the volume information, but still require invasive LV pressure measurements. In a study by Schmitt et al. (2009), invasive LV pressure data were combined with two-dimensional phase-contrast MRI (2D PC-MRI) data for assessing the LV ESPVR and EDPVR in swine. In conventional clinical practice, the inability to make these accurate measurements has led to substitution with load-dependent surrogate markers of LV systolic and diastolic function such as ejection fraction and mitral inflow patterns derived from Doppler echocardiography and 2D PC-MRI (el-Said et al., 1994; Iwakami et al., 1996; Paelinck et al., 2004). The limitations in accuracy and performance of load-dependent measures for defining actual LV contractility and compliance are well recognized, and a source of uncertainty and potential error in patient care (Thomas and Popović, 2006).

The response of LV contractility and relaxation across a physiologically relevant range of load and heart rate is more predictive of disease than isolated resting measurements. Clinically, stress testing to expose abnormalities in cardiac function is often performed with infusion of dobutamine, a synthetic catecholamine with a variable effect on the α- and β-adrenoreceptors in the cardiovascular system (Tuttle and Mills, 1975; Barnett, 1989). In the healthy heart, the combined stimulation of these adrenoreceptors is expected to improve left ventricular contractility (inotropic effect), increase heart rate (chronotropic effect), and accelerate left ventricular relaxation (lusitropic effect). Hemodynamic responses to dobutamine are variable even amongst healthy subjects, but can include an increase in cardiac output and a decrease in total peripheral vascular resistance (Liang and Hood, 1979; Binkley et al., 1991; Parker et al., 1991).

Mathematical modeling to characterize left ventricular systolic and diastolic properties can incorporate the influence of multiple cardiovascular factors that contribute to the variable response to dobutamine among individual subjects. Mechanistic, physiologically based models permit data assimilation for identifying subject-specific parameters and estimating hemodynamic variables that are difficult to obtain non-invasively. Emerging imaging techniques have enabled characterization of the heart from anatomy to several functional measures including heart motion, myofiber architecture, blood flow, and even metabolism (Lamata et al., 2014). This has made possible to personalize three-dimensional models of cardiac biomechanics using invasive and/or non-invasive measurements from different modalities (Smith et al., 2011; Trayanova et al., 2011). In the context of assessing systolic and diastolic ventricular function such models can be applied to, for instance, generate left ventricular pressure-volume loops and ventricular pressures (Krishnamurthy et al., 2013) and to estimate regional cardiac contractility (Chabiniok et al., 2012).

Lumped parameter representations are often used as boundary conditions for higher dimensional models (Gurev et al., 2011), but can also be used individually to describe global hemodynamics (Heldt et al., 2002; Zhong et al., 2005; Ellwein et al., 2008). In this type of models, the contractility of the heart chambers is typically represented using a mathematical description of the time-varying elastance (Suga et al., 1973; Stergiopulos et al., 1996). Lumped parameter models are relatively simple and less computationally expensive than three-dimensional approaches, which makes them suitable for real-time simulation in the clinical setting.

In a previous study, we developed an approach combining non-invasive measurements, including three-directional three-dimensional cine phase-contrast magnetic resonance imaging (4D Flow MRI) data (Dyverfeldt et al., 2015), and a lumped parameter model of the cardiovascular system to obtain personalized parameters characterizing global hemodynamics (Casas et al., 2017). The model presented in our previous study, which includes the influence of the proximal aortic and peripheral vasculature, can more broadly represent the total cardiovascular response to changes in loading conditions, and potentially provide a more accurate estimate of load-independent contractility and relaxation parameters. However, this approach has so far been tested only in healthy subjects and its applicability under stress conditions has not yet been investigated.

In this work, we aim to investigate whether our modeling approach based on non-invasive data can be used to quantify, on an individual basis, changes in cardiovascular function during dobutamine stress, with particular focus on load-independent parameters of LV contractility and relaxation that conventionally require specialized invasive measurements. In addition, with this investigation we aim to test the applicability of the modeling approach beyond the resting operating conditions for which it was initially developed. This represents a key step toward assessing the usefulness of the developed approach in the clinical setting.

## Materials and methods

### Study population

We personalized parameters in the computational model using non-invasive measurements from subjects without a known cardiovascular disease, acquired prior to and during the infusion of dobutamine. Inclusion criteria were no history of cardiovascular disease and no medication for cardiovascular disease. The exclusion criteria were arterial hypertension (values of arterial blood pressures ≥140/90 mmHg; Mancia et al., 2013), absence of normal ventricular size, wall thickness or wall motion based on the morphological balanced steady-state free precession (bSSFP) images at rest, acute coronary artery disease, severe aortic stenosis, hypertrophic obstructive cardiomyopathy and heart failure NYHA class superior to II. Reference values for the EDV (Maceira et al., 2006) were used to assess if subjects had normal ventricular size. Wall thickness and wall motion were visually assessed on the bSSFP images by a clinician with 15 years of experience in cardiovascular imaging. Fourteen subjects were enrolled in the study, although five subjects were excluded based on data quality. The resulting study group consisted of nine subjects (seven females), with an age of 29 ± 11 years (range 20–56 years), height 172 ± 9 cm (range 156–184 cm), and weight 67 ± 9 kg (range 52–85 kg).

The subjects were recruited using public advertisement on bulletin boards at the Linköping University Hospital. This study was carried out in accordance with the recommendations of the Regional Ethical Review Board in Linköping. All subjects gave written informed consent in accordance with the Declaration of Helsinki. The protocol was approved by the Regional Ethical Review Board in Linköping (Dnr 2014/114-31).

### Study protocol

The subjects underwent an MRI protocol consisting of two acquisitions, one acquisition at rest and a second acquisition during the infusion of dobutamine. The dobutamine infusion protocol was initiated after the acquisition of 4D Flow MRI data performed at rest. Dobutamine was administered intravenously starting at a dose between 5 and 10 μg/kg/min, and the dose was increased or decreased in intervals of ~2 min depending on the observed effect on the subject's heart rate and arterial pressure. The target heart rate was 50% higher than the subject's rest heart rate, in order to achieve heart rates in the range of those from previous studies on stress testing with cardiac magnetic resonance (CMR) (Pennell et al., 1992; van Rugge et al., 1994; Roest et al., 2001; Steding-Ehrenborg et al., 2013). Heart rate was monitored continuously throughout the study. The blood pressures were measured at rest and during the administration of dobutamine, approximately every second minute, using a cuff sphygmomanometer. The dobutamine infusion was maintained until the scans in the second MRI acquisition were completed.

### MRI examinations and data processing

All MRI examinations were performed on a clinical 3T Philips Ingenia scanner (Philips Healthcare, Best, the Netherlands). During the examination, we acquired morphological cine bSSFP images and 4D Flow MRI data.

Morphological bSSFP images included three- and four-chamber long-axis (LAx) images and a stack of SAx images. During the scan performed after the infusion of dobutamine, only the three-chamber image and the SAx stack were acquired. The images were acquired during end-expiratory breath holds using the following settings: echo time 1.4 ms, repetition time 2.8 ms, flip angle 45° and slice thickness 8 mm. The bSSFP images were reconstructed into 30 time frames. The SAx images were reconstructed with a pixel size of 0.9 × 0.9 mm^2^ and the LAx images 0.83 × 0.83 mm^2^.

4D Flow MRI data were acquired during free breathing, using a navigator gated gradient-echo pulse sequence with interleaved three-directional flow-encoding and retrospective vector cardiogram controlled cardiac gating (Dyverfeldt et al., 2008; Eriksson et al., 2010). Sequence parameters were: velocity encoding (VENC) 140 cm/s, flip angle 5°, echo time 3.0 ms, repetition time 5.2 ms, parallel imaging (SENSE) speed up factors of 3 (AP direction) and 1.6 (RL direction), k-space segmentation factor 2 and elliptical k-space acquisition. The spatial resolution was 2.8 × 2.8 × 2.8 mm^3^ and the temporal resolution 41.6 ms. This resulted in a scan time of ~7–8 and 10–15 min excluding and including navigator efficiency, respectively, for a heart rate of 60 bpm. Following acquisition, the data were retrospectively reconstructed into 40 time frames and corrected for concomitant gradient fields on the scanner. Subsequently, the data were transferred to an offline station for post-processing and analysis. Post-processing was performed using in-house developed software written in Matlab (The Mathworks Inc., Natick, Massachusetts, USA). This included correction for background errors using a weighted second-order polynomial fit to static tissue in the thorax (Ebbers et al., 2008) and for phase wraps by the use of a temporal algorithm (Xiang, 1995).

### Personalization of model parameters

#### Lumped-parameter model of the cardiovascular system

**Figure 1:**
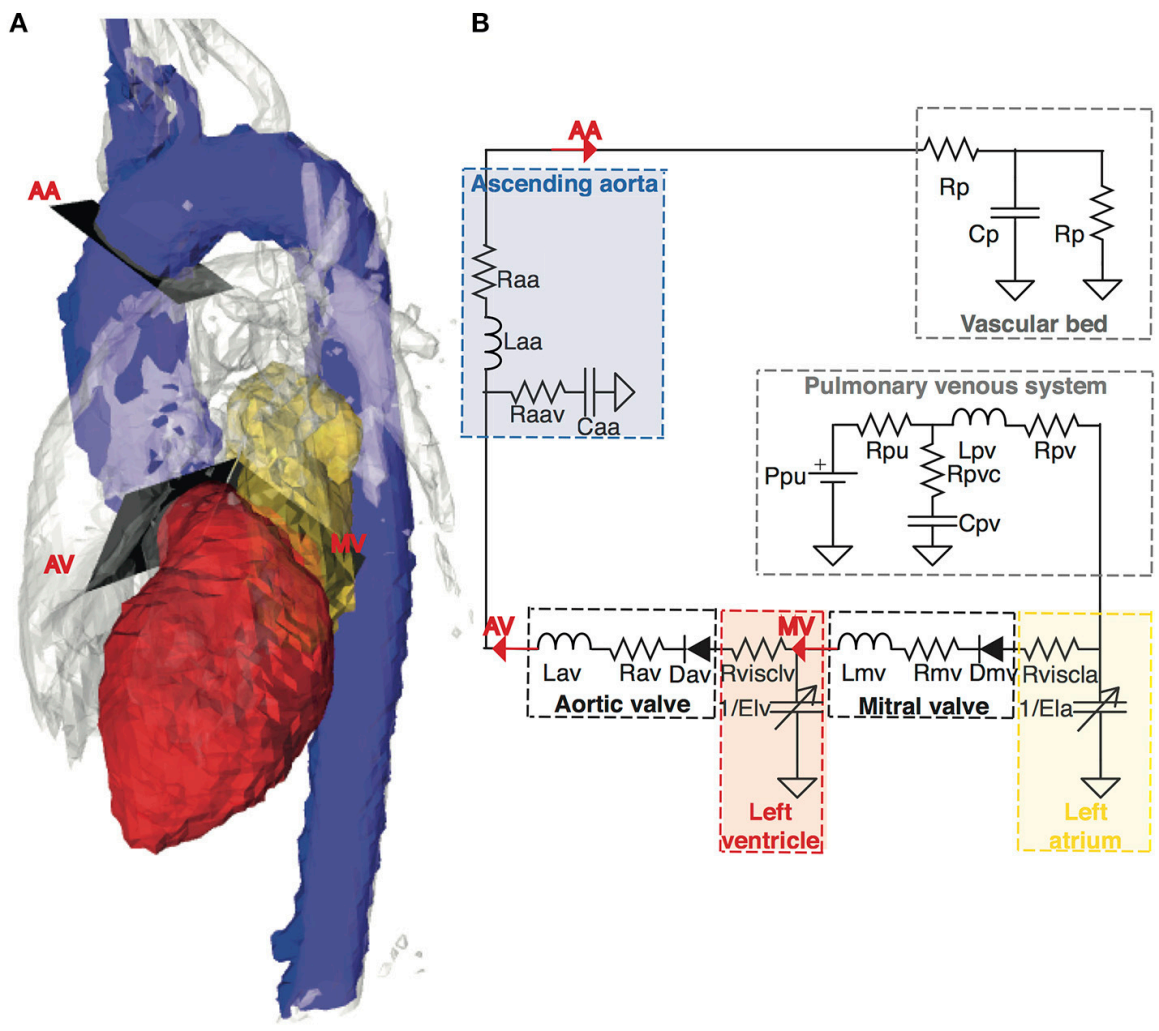
Anatomical configuration and equivalent lumped parameter model of the cardiovascular system. **(A)** Anatomy of a representative subject shown as a 4D PC-MR angiogram at peak systole, including segmentations of anatomical regions of interest for the model: the left atrium (yellow), the left ventricle (red), and the aorta (blue). The analysis planes used for extracting subject-specific input flows for the model are located at the mitral valve (MV), aortic valve (AV), and ascending aorta (AA). **(B)** A schematic representation of the lumped parameter model. The building blocks corresponding to the different anatomical compartments are highlighted in dashed lines and color-coded according to the anatomical representation in **(A)** Descriptions of the parameters in the model can be found in Supplementary Table S1.

We implemented a reduced version of our previous model of the heart and the systemic circulation (Casas et al., 2017), in order to focus on the physiology of the left ventricle and the systemic vasculature. The model used in this study included three compartments: the pulmonary venous system, the left side of the heart (including the left atrium, the mitral valve, the left ventricle, and the aortic valve) and the systemic arterial system. The model of the systemic arterial circulation was reduced to include the ascending aorta and a single compartment representing the vessels in the systemic circulation, down to the peripheral vascular bed. A schematic representation of the cardiovascular model is shown in Figure 1.

To characterize the contractile state of the heart chambers over the cardiac cycle, we used the concept of time-varying elastance formalized by Suga et al. (1973). This is a well-established formulation to describe the filling and the contraction patterns of the chambers. The time-varying elastance relates chamber pressure and volume along the cardiac cycle, as given by:

(1) $$\begin{array}{l} E\left(t\right)=\frac{P\left(t\right)}{V\left(t\right)-V_{o}}\text{(mmHg/mL)} \end{array}$$

where *P*(*t*) is the chamber pressure and *V*(*t*) is the volume. *V*_*o*_ is the unstressed volume, defined as the volume-axis intercept of the end-diastolic relationship with the volume axis.

**Figure 2:**
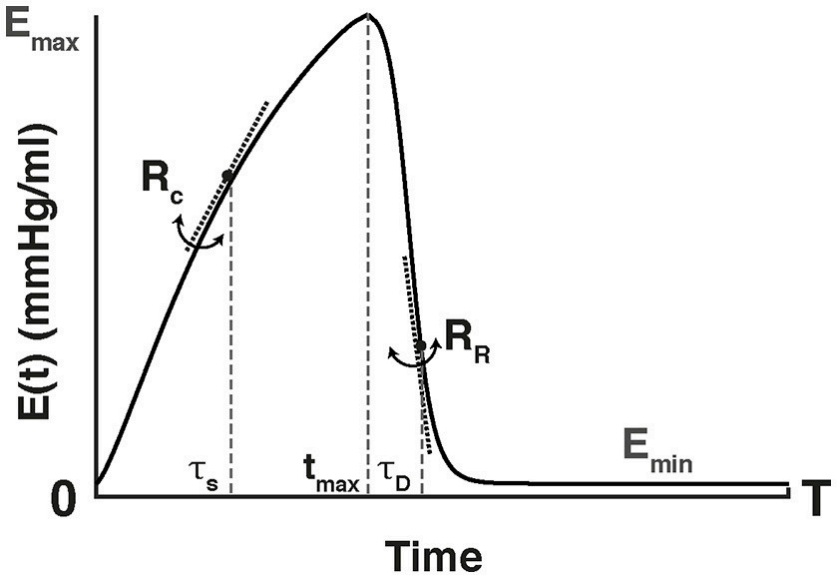
Schematic representation of the time-varying elastance curve, E(t), used to describe the heart chambers. The elastance values range from the end-systolic, maximal elastance (*E*_*max*_) to its minimal value (*E*_*min*_) over the duration of the cardiac cycle (T). The maximal elastance occurs at the time of end-systole (*t*_*max*_). The time instant zero corresponds to the onset of isovolumic contraction. The shape is determined by the ascending slope or contraction rate (*R*_*C*_), the descending slope or relaxation rate (*R*_*R*_) and the systolic (α_*s*_), and diastolic (α_*D*_) time constants. These constants determine the length of systole and diastole relative to the cardiac cycle duration (T), thereby controlling *t*_*max*_.

The shape of the elastance curve was approximated by a Double-Hill function (Stergiopulos et al., 1996; Mynard et al., 2012):

(2) $$\begin{array}{l} E\left(t\right)=\alpha (E_{\max}-E_{\min})\left(\frac{{\left(\frac{t}{\alpha_{S}T}\right)}^{R_{C}}}{1+{\left(\frac{t}{\alpha_{S}T}\right)}^{R_{C}}}\right)\left(\frac{1}{1+{\left(\frac{t}{\alpha_{D}T}\right)}^{R_{R}}}\right)\\ \;+\;E_{\min}\text{(mmHg/mL)}. \end{array}$$

where *T* is the cardiac cycle length, and *E*_max_ and *E*_min_ are the values of maximal and minimal elastance (i.e., stiffness of the chamber). For the left ventricle, *E*_max_ corresponds to the end-systolic elastance and *E*_min_ characterizes the passive diastolic elastance of the chamber. A representative cardiac elastance curve is illustrated in Figure 2. The dimensionless parameters α_*S*_, α_*D*_, *R*_*C*_, and *R*_*R*_ determine the shape of the curve within each cardiac period. The rate of chamber contraction, reflected as the slope of the ascending part of the curve, is represented by the parameter *R*_*C*_. Similarly, *R*_*R*_ corresponds to the rate of chamber relaxation (i.e., the steepness of the descending part of the curve). The systolic and diastolic time constants (τ_*S*_ and τ_*D*_, respectively) are defined as a function of *T* and the dimensionless shape parameters α_*S*_ and α_*D*_ (τ_*S*_ = α_*S*_ · *T* and τ_*D*_ = α_*D*_ · *T*). α_*S*_ and α_*D*_ determine the length of systole and diastole relative to each cardiac cycle each cardiac cycle, thereby controlling the time of end-systole (*t*_max_). An increase in α_*D*_ and/or a decrease in α_*s*_ yield an increase in *t*_max_ (i.e., a reduction of the duration of diastole), and vice versa. In the following, we refer to α_*S*_ and α_*D*_ as systolic and diastolic time constant shape parameters, respectively. The scaling factor α ensures a maximal elastance of *E*_max_.

The pressure gradient across the aortic valve was modeled using the formulation of the net pressure gradient introduced by Garcia et al. (2005), which takes into consideration the pressure recovery phenomenon (Baumgartner et al., 1999). Using this formulation, the pressure gradient is computed as a function of the transvalvular flow rate, the effective orifice area (EOA) of the valve and the cross-sectional area of the aorta. Pressure recovery was not considered at the mitral valve, as its effect on the transmitral pressure gradient is negligible in healthy subjects (Vandervoort et al., 1995).

Vessel segments in the systemic circulation were modeled using a combination of two resistances (*R* and *Rv*), a compliance (*C*), and an inductance (*L*). In each segment, the resistance *R* and the inductance *L* represent frictional losses and mass flow inertia, respectively, while the combination of compliance *C* and resistance *Rv* represent the viscoelastic properties of the vessel wall (Sun et al., 1995). The systemic vascular bed, down to the capillaries, was represented using a three-element viscoelastic Windkessel model (Burattini and Natalucci, 1998).

The model equations were implemented in Matlab Simscape 2017a (The Mathworks Inc., Natick, Massachusetts, USA). Details on the simulation settings for solving the model equations are described in Casas et al. (2017).

#### Subject-specific input measurements

For each subject in the study, we computed all model-based parameters at rest and following the infusion of dobutamine. These parameters were estimated based on subject-specific measurements acquired at rest and after dobutamine administration. The subject-specific inputs include the duration of the cardiac cycle, MRI-derived measurements characterizing the morphology and function of the left ventricle and the aortic valve, as well as volumetric flow waveforms from three selected sites in the systemic circulation. These three sites correspond to the mitral valve, the aortic valve and the ascending aorta, upstream from the brachiocephalic trunk (MV, AV, and AA in Figure 1, respectively). The analysis to obtain the MRI-derived input measurements was performed by a single observer. These imaging data were combined with non-invasive cuff systolic and diastolic pressure measurements in the brachial artery.

The length of the cardiac cycle (T) was derived as the average cardiac cycle duration throughout the 4D Flow MRI acquisition. Estimation of the left ventricular elastance requires computing the left ventricular end-systolic volume (LVESV). This volume was calculated by manual delineation of the endocardial border, including the papillary muscles, in the short-axis stack at the time of end systole. In estimating the EOA of the aortic valve, the stroke volume (SV) was computed by integrating the volumetric flow curve at the aortic valve over the entire cardiac cycle. The velocity-time integral of the transvalvular flow (VTI) was calculated using the instantaneous velocity at the center of the valve. The cross-sectional area of the aorta was measured at the time of peak systole, distal to the coronary artery ostia.

Transmitral (MV) and transaortic (AV) valvular flows were extracted using a semi-automatic retrospective valve tracking approach (Westenberg et al., 2008), in order to accurately locate the analysis plane based on the position of the valve annulus. The approach requires two manually placed input points to identify the valve annulus in the end-diastolic time frame. The analysis plane at the ascending aorta was placed manually, using a 4D PC-MR angiogram generated from the 4D Flow MRI data for anatomical orientation. The volume flow through each analysis plane was calculated by manual delineation of the valve orifice or the vessel contour for every time frame along the cardiac cycle (Stalder et al., 2008). Details on the calculation of the volumetric flow waveforms are given in Casas et al. (2017).

Besides the subject-specific measurements involved in model personalization, we derived additional measurements characterizing peripheral hemodynamic responses to dobutamine infusion in each subject. The systemic vascular resistance (SVR) was computed as the mean arterial blood pressure (MAP) divided by the cardiac output (CO), as described elsewhere (Casas et al., 2017). The total systemic arterial compliance was calculated as the ratio between the SV and the pulse pressure (PP), computed as the difference between systolic blood pressure (SBP), and diastolic blood pressure (DBP) (Otsuki et al., 2008).

#### Subject-specific parameter estimation

The cardiovascular model consists of 40 parameters. Of these parameters, 6 represent the pulmonary venous system, 20 characterize the heart chambers, 5 the heart valves and 7 the systemic vessels. The cardiac cycle length (T), and the density of blood (ρ) are also defined as parameters. Twenty three parameters were adjusted to be subject-specific, while the remaining 17 were defined based on literature values from previous studies (Sun et al., 1995; Heldt et al., 2002; Garcia et al., 2012; Mynard et al., 2012; Broomé et al., 2013). The parameters established according to literature along with their corresponding values are given in Supplementary Table S1. The choice of parameters to be adjusted for personalization was based on the availability of *in vivo* measurements, as well as the results from the identifiability analysis performed in our previous study (Casas et al., 2017). In particular, structurally non-identifiable parameters and those describing the pulmonary vessels were set to literature values. Adjustable parameters were personalized based on measures derived from the *in vivo* data, or using nonlinear optimization to obtain the best agreement between the model-based and the measured flow waveforms. The number of parameters estimated using the nonlinear optimization routine was 20. A detailed description of the approach for tuning the subject-specific parameters, including the nonlinear optimization routine, is given in Casas et al. (2017).

### Image-processing tools

The left ventricular segmentations were performed using the freely available segmentation software Segment version 1.9 (Medviso, Lund, Sweden) (Heiberg et al., 2010). The commercially available software Ensight (CEI Inc., NC, USA) was used for flow visualizations, creation of the 4D PC-MR angiograms and placement of the analysis planes at the locations of interest. Retrospective valve tracking and flow quantification at the analysis planes were carried out using in-house software written in Matlab (The Mathworks Inc., Natick, Massachusetts, USA).

### Statistical analysis

Statistical analysis was performed using SPSS Statistics v.23.0 (IBM Corp., Armonk, NY, USA). We performed non-parametric Wilcoxon signed-ranked tests to assess the differences between variables at rest and under dobutamine stress. A *p* < 0.05 was considered significant. All data are presented as mean ± standard deviation (SD).

## Results

All subjects completed the study according to the protocol and no complications were seen in relation to the infusion of dobutamine. Across all subjects, the peak dose infused during the scan time was 15 ± 6 μg/kg/min (range 10–25 μg/kg/min). The maximal peak dose of 25 μg/kg/min was reached in two subjects. Table 1 presents demographic data and the dobutamine peak doses administered during the MRI examination for each subject in the study.

**Table 1:** Demographic data and peak dobutamine doses during the MRI examination for all nine subjects in the study.

**Subject number**	**Age range (years)**	**Weight range (kg)**	**Height range (cm)**	**Peak dose during the MRI examination (μg/kg/min)**
1	30–39	60–69	170–179	15
2	20–29	70–79	180–189	10
3	20–29	60–69	170–179	10
4	20–29	70–79	170–179	25
5	20–29	60–69	160–169	12
6	20–29	60–69	170–179	12
7	20–29	80–89	170–179	25
8	30–29	50–59	150–159	10
9	50–59	60–69	160–169	15

*The age, weight, and height of each subject are presented as a range*.

### MRI-derived indices of cardiovascular function

**Figure 3:**
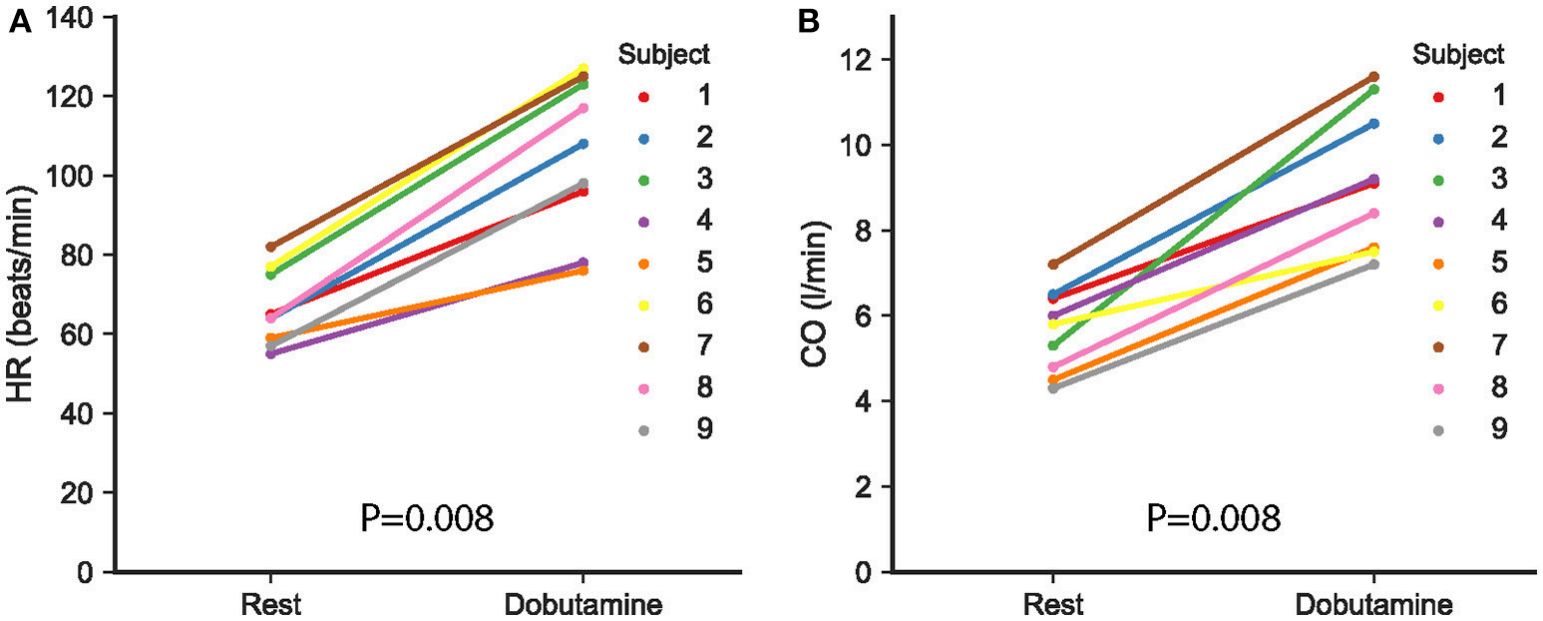
**(A)** Heart rate (HR) and **(B)** cardiac output (CO), as derived from the MRI measurements, at rest and following the infusion of dobutamine in the nine study subjects. Each subject is represented with different color, as indicated in the figure. A value of *p* < 0.05 was considered significant.

Individual changes in heart rate (HR) and cardiac output (CO) induced by the infusion of dobutamine are shown in Figure 3. Dobutamine increased heart rate and cardiac output in all subjects, yielding significant differences from 66 ± 9 to 105 ± 19 beats/min (*p* = 0.008) and 5.6 ± 1 to 9.2 ± 1.7 l/min (*p* = 0.008), respectively (Table 2).

**Table 2:** MRI-derived indices of cardiac function extracted at rest and following the infusion of dobutamine.

	**Rest**	**Dobutamine**	***p***
HR (beats/min)	66 ± 9 (55–82)	105 ± 19 (76–127)	0.008^*^
CO (l/min)	5.6 ± 1(4.3–7.2)	9.2 ± 1.7 (7.2–11.6)	0.008^*^
SBP (mmHg)	115.5 ± 12.8 (98–137)	139.2 ± 19.9 (119–186)	0.013^*^
MAP (mmHg)	86.4 ± 10.2 (76.2–102.3)	98.3 ± 11 (87.1–117.8)	0.066
DBP (mmHg)	66 ± 10.3 (56–85)	63.7 ± 8.1 (53–77)	0.48
LVESV (mL)	70.3 ± 18.9 (45.4–103.6)	48.9 ± 24 (19–88.2)	0.015^*^
LVEDV (mL)	153.5 ± 27.9 (123.3–199.7)	141.8 ± 41.5 (81–217.5)	0.17
SV (mL)	85.4 ± 14 (70.3–108.8)	88.9 ± 17.8 (59–118.5)	0.51
EF (%)	56 ± 8 (47–71)	65 ± 10 (53–82)	0.066
SVR (mmHg·s/mL)	0.97 ± 0.23 (0.69–1.46)	0.66 ± 0.14 (0.46–0.9)	0.008^*^
CT (mL/mmHg)	1.9 ± 0.52 (1.2–2.8)	2.1 ± 0.41 (1.3–2.6)	0.37

*Values are presented as mean ± standard deviation, with ranges given in parentheses. HR, heart rate; CO, cardiac output; SBP, systolic blood pressure; MAP, mean arterial pressure; DBP, diastolic blood pressure; LVESV, left ventricular end-systolic volume; LVEDV, left ventricular end-diastolic volume; SV, stroke volume; EF, ejection fraction; SVR, systemic vascular resistance; CT, total systemic arterial compliance*.

* *p < 0.05 vs. rest*.

**Figure 4:**
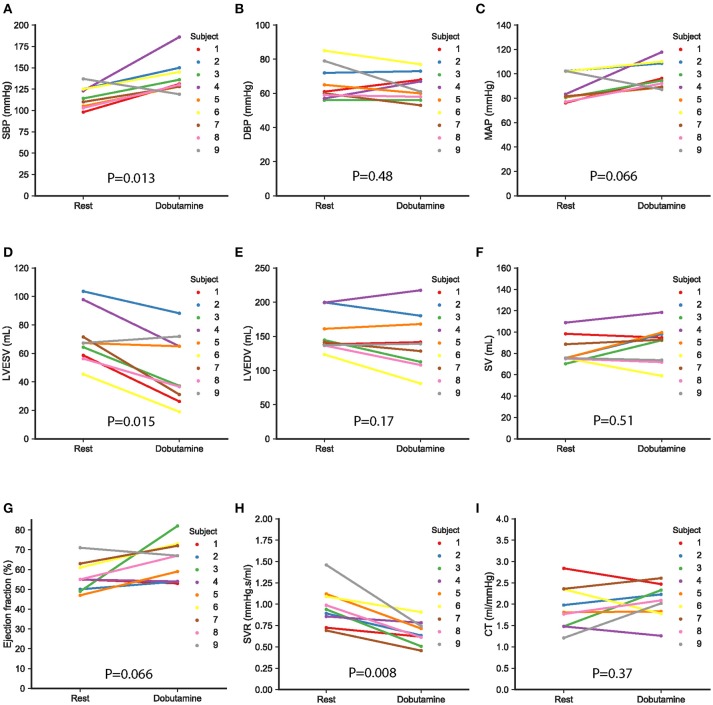
Arterial pressures, ventricular volumes, and systemic resistance and compliance at rest and following the infusion of dobutamine in the nine study subjects. Values are derived from the MRI data and the non-invasive cuff pressure measurements **(A–C)** show dobutamine-induced changes in systolic blood pressure (SBP), diastolic blood pressure (DBP), and mean aortic pressure (MAP), respectively. **(D–F)** illustrate changes in left ventricular end-systolic volume (LVESV), left ventricular end-diastolic volume (LVEDV), and stroke volume (SV). Changes in ejection fraction (EF), systemic vascular resistance (SVR), and total arterial compliance are shown in **(G–I)**, respectively. Each subject is represented with a different color, as indicated in the figure. A value of *p* < 0.05 was considered significant.

Figure 4 depicts individual changes with dobutamine in additional indices derived from the MRI data and the non-invasive cuff pressure measurements. The results are summarized in Table 2. SBP increased significantly (Table 2, Figure 4A), while non-significant changes were found for DBP and MAP (Table 2, Figures 4B,C). Left ventricular ESV decreased significantly (Table 2, Figure 4D). No significant differences were found in left ventricular EDV, SV, or EF (Table 2, Figures 4E–G). Systemic vascular resistance (SVR) decreased significantly in response to dobutamine. On the contrary, no significant changes were seen in total systemic arterial compliance (Table 2, Figures 4H,I).

### Model-based parameters and 4D flow MRI volumetric flow waveforms

**Figure 5:**
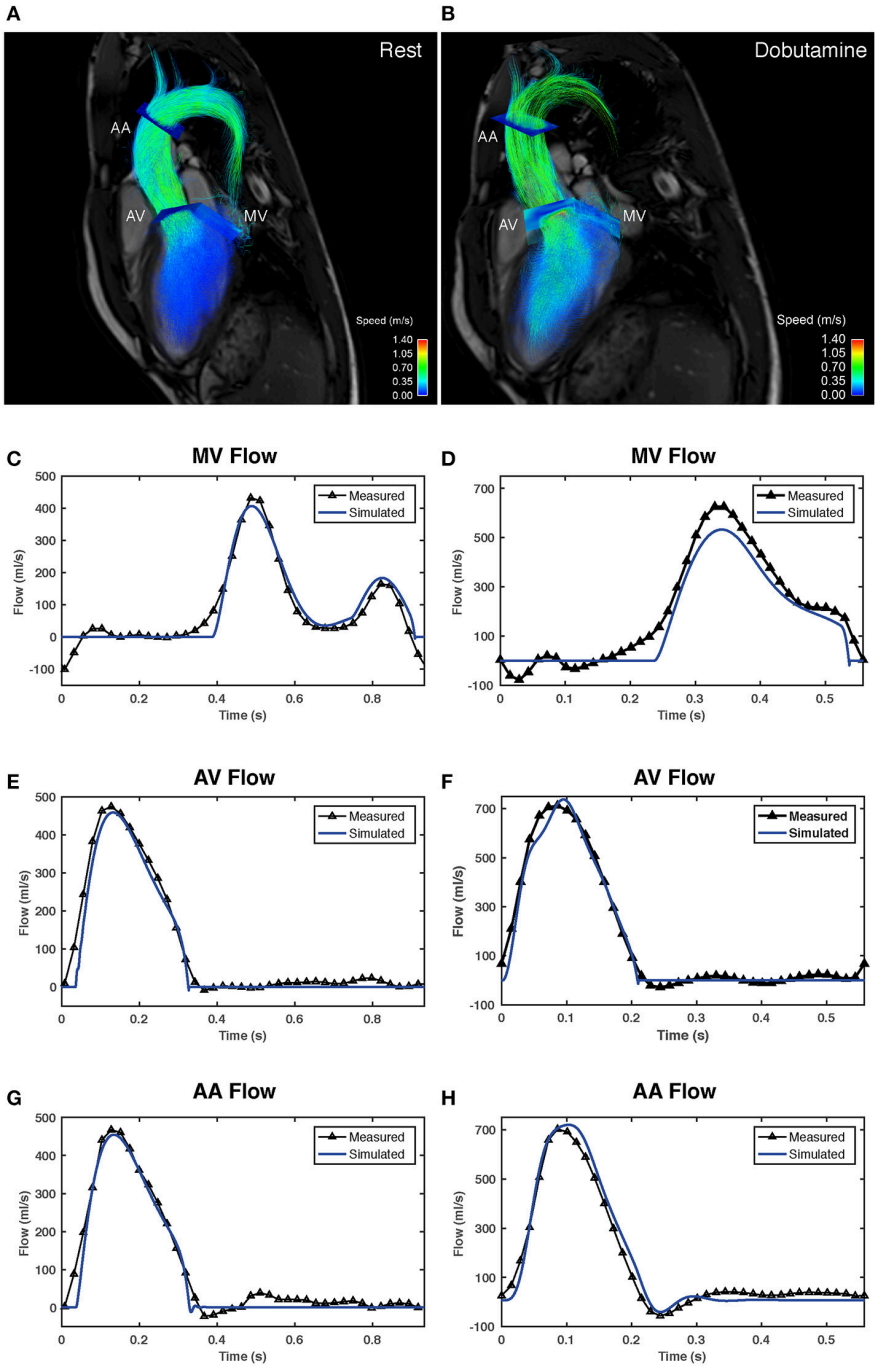
Comparison between volumetric flow waveforms derived from the 4D Flow MRI data and model-based waveforms obtained after subject-specific fitting in one of the study subjects (subject 2). **(A,B)** show flow visualizations in the left heart and the ascending aorta. The visualizations in **(A,B)** correspond to the scans acquired at rest and following the infusion of dobutamine, respectively. Pathlines covering the complete heart cycle are overlaid onto three-chamber anatomical images. Pathlines are color-coded according to speed. The visualization includes the analysis planes used to extract the volumetric flow waveforms. **(C,E,G)** show a comparison between the measured (black) and the model-generated (blue) flow waveforms in the mitral valve (MV), the aortic valve (AV), and the ascending aorta (AA) at rest. Comparisons between estimated and measured flow waveforms after dobutamine administration for the same subject are shown in **(D,F,H)**.

Figure 5 shows the measured and the model-based volumetric flow waveforms for a representative subject at baseline (Figures 5C,E,G) and following the infusion of dobutamine (Figures 5D,F,H). The subject-specific fitting was performed successfully in all nine subjects included in the study. At rest, the root mean square error (RMSE) between the measured and the model-based flow waveforms across the nine study subjects was 25 ± 6.6 ml/s at the mitral valve, 21 ± 5.1 ml/s at the aortic valve and 22.2 ± 4.9 ml/s at the ascending aorta. Under dobutamine stress, the RMSE values between the waveforms at these locations were 53 ± 18.5, 49.9 ± 22.7, and 37±13 ml/s, respectively. The RMSE values for every subject are listed in Supplementary Table S2. Analysis of the 4D Flow MRI-derived flow waveforms revealed a significant increase in early (E) peak flow with dobutamine, from 427.1 ± 88.2 to 534.7 ± 128.8 mL/s (*p* = 0.011). Dobutamine administration also increased the late (A) peak flow from 164.6 ± 45.5 to 266.4 ± 94.5 mL/s (*p* = 0.008). Furthermore, we observed that the E and A waves of the mitral flow waveform progressively merged with increasing heart rates, as illustrated in Supplementary Figure S1. Across all subjects, there was no significant change in E/A ratio with dobutamine (2.9 ± 1.2 vs. 2.4 ± 1.6, *p* = 0.14). Supplementary Figure S1 gives an overview of the mitral flow patterns, at rest and under dobutamine stress, for all nine subjects in the study. The peak flow at the aortic valve increased significantly from 405.4 ± 67.8 to 659.3 ± 86.6 mL/s (*p* = 0.008), as did the peak flow at the ascending aorta (366.8 ± 75.4 to 595.4 ± 94.7 mL/s, *p* = 0.008).

**Figure 6:**
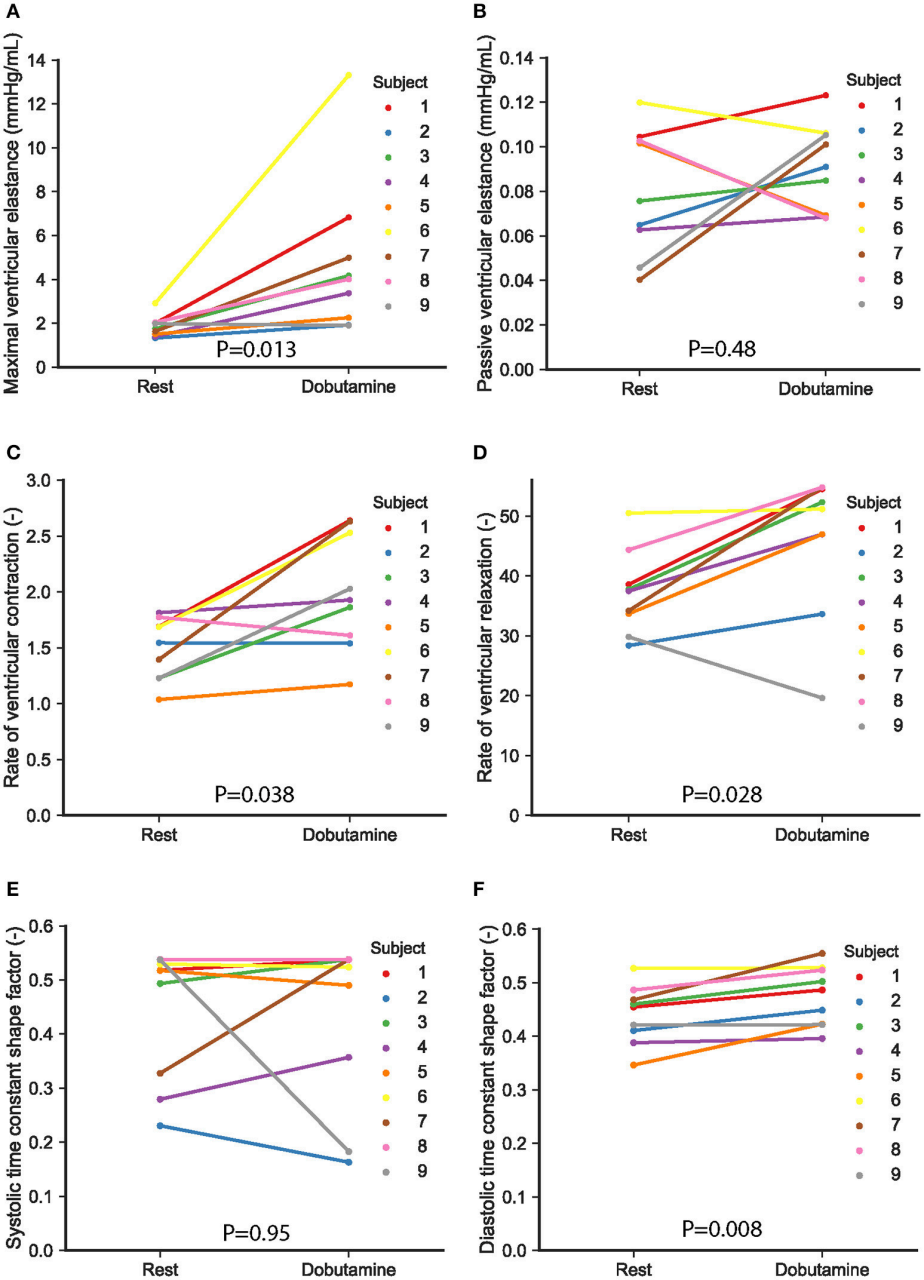
Model-based parameters characterizing the left ventricular time-varying elastance in the nine study subjects. Changes with dobutamine in maximal (*E*_*max,LV*_) and passive diastolic elastance of the left ventricle (*E*_*min,LV*_) are shown in **(A,B)**. Individual changes in parameters describing the shape of the elastance are given in **(C–F)**, as follows: **(C)** rate of ventricular contraction *R*_*C,LV*_, **(D)** rate of ventricular relaxation *R*_*R,LV*_, **(E)** ventricular systolic time constant shape parameter α_*S,LV*_, and **(F)** ventricular diastolic time constant shape parameter α_*D,LV*_. Each subject is represented with a different color, as indicated in the figure. A value of *p* < 0.05 was considered significant.

The changes with dobutamine in the parameters describing the time-varying elastance are illustrated in Figure 6. There was a significant increase in left-ventricular maximal elastance (*E*_*max, LV*_) from 1.8 ± 0.5 to 4.7 ± 3.6 mmHg/mL (*p* = 0.013), as seen in Figure 6A. The passive left-ventricular elastance (*E*_*min, LV*_) did not change (*p* = 0.48; Figure 6B). Inotropic stimulation with dobutamine also increased significantly the rate of ventricular contraction, *R*_*C, LV*_, from 1.5 ± 0.3 to 2 ± 0.5 (*p* = 0.038; Figure 6C). In addition, dobutamine induced a significant acceleration in ventricular isovolumic relaxation, as indicated by the change in *R*_*R, LV*_, from 37.2 ± 6.9 to 46.1 ± 12 (*p* = 0.028; Figure 6D)

The increase in heart rate due to dobutamine infusion resulted in shortening of both the systolic and diastolic phases, with a moderately greater reduction in the duration of diastole in relation to systole. The percentage of diastolic duration relative to the cardiac cycle length was calculated based on the time of end-systole (*t*_*max*_), extracted from the estimated time-varying elastance curve. Among all subjects, the decrease in this percentage was between 1 and 10%, with larger decreases in the diastolic contribution associated with higher heart rates. Shortening of diastole in relation to the cardiac cycle length was reflected in the elastance timing parameters by a significant increase in the diastolic time constant shape parameter, α_*D, LV*_, from 0.4 ± 0.05 to 0.5 ± 0.05 (*p* = 0.008; Figure 6F). This resulted in a decrease of the elastance diastolic time constant from 0.4 ± 0.04 s to 0.3 ± 0.03 s (*p* = 0.008). Analysis of model-based parameters characterizing the vascular system revealed a non-significant increase in the compliance of the ascending aorta, *C*_*aa*_, from 0.11 ± 0.06 to 0.16 ± 0.1 mL/mmHg (*p* = 0.11).

## Discussion

We evaluated a previously developed imaging-modeling approach for quantification of load-independent parameters of LV contractility and relaxation. An advantage of this approach is that it allows the estimation of physiological parameters, which typically require invasive measurements, in a non-invasive fashion. The evaluation was performed on a cohort of nine subjects without a known heart disease, both at rest, and under stress conditions induced by the infusion of dobutamine. Our results indicate that the approach is suited for both description of the experimental data and determination of parameters characterizing LV systolic and diastolic function across a physiologically relevant range of heart rates and loading conditions. This demonstrates that our approach has the ability to describe operating conditions for which it was not originally developed and serves to validate the realism of the model. This is therefore a key stepping stone toward eventual clinical implementation.

Several studies have investigated the effects of clinically induced stress on cardiovascular function based on non-invasive indices derived from Doppler ultrasound or 2D PC-MRI measurements, such as left ventricular volumes and mitral inflow patterns. In line with our results, these studies have reported a decrease in left ventricular ESV (Steding-Ehrenborg et al., 2013), enhanced mitral peak E and A flows (el-Said et al., 1994; Kilner et al., 1997; Paelinck et al., 2004). In addition, like others (Kilner et al., 1997; Chung et al., 2004; Paelinck et al., 2004), we found a progressive merging of mitral E and A waves as a result of increasing heart rates during stress conditions.

The model-based parameters revealed subject-specific increases in contractility as indicated by the increase in rate of left ventricular contraction under stress conditions, as well as an enhancement of diastolic function by faster early relaxation. The latter was demonstrated by an increase in the rate of left ventricular relaxation. These results are consistent with previous studies reporting an increase in these parameters based on invasive measurements (Karliner et al., 1977; Little et al., 1987; Myreng and Smiseth, 1990; Udelson et al., 1990). Similar to previous observations from mitral inflow patterns (Kilner et al., 1997; Paelinck et al., 2004), the systolic and diastolic time constant shape parameters derived from the elastance function indicate the shortening of diastolic length at higher heart rates. One of the study participants (subject 9) exhibited a response to dobutamine with changes in hemodynamic variables different from the average changes observed in the study group, as seen in Figure 4. For this specific subject, the elastance model parameters revealed a decrease in both maximal ventricular elastance and the relaxation rate. These results contrast with the behavior of the parameters found in the rest of study subjects. A possible explanation for this discrepancy is the presence of ventricular dysfunction, which was unmasked at the higher rates achieved with the stress protocol.

In the present study, we estimated the maximal elastance of the left ventricle using a single-beat approach, assuming a volume intercept of 10 mmHg as defined in previous studies (Mynard et al., 2012). Alternatively, this assumption can be avoided by using single-beat methods that involve approximation of the pre-ejection period to total systolic period. These methods have been shown to provide a better estimation of the maximal elastance, as measured by conventional pressure- volume measurements with varying loading conditions (Senzaki et al., 1996), including variations in inotropic state and different cardiovascular diseases. In our study, a volume intercept of 10 mmHg might result in some overestimation of the actual maximal elastance considering the decreased LVESV values with dobutamine, and therefore this should be taken into consideration when interpreting the results from the model. Accurate estimations of the time-varying elastance also require precise measurements of the LVESV. Currently, CMR is considered the gold standard for measuring ventricular volumes with regard to reproducibility and accuracy (Danilouchkine et al., 2005). The left ventricular segmentations performed in this study were based on short-axis morphological images, a well-validated approach (Sievers et al., 2004) that is generally incorporated into the clinical CMR protocol. To avoid inaccuracies in the estimated LVESV, the short-axis images acquired during the stress protocol were examined and acquisition was repeated, if necessary, to ensure sufficient image quality.

Our results showed no significant differences between passive diastolic compliance at rest and during stress. Schmitt et al. (2009) reported an increase with dobutamine in diastolic compliance, calculated as an exponential fit of the EDPVR measured using combined catheter and CMR measurements in swine. A possible explanation for this discrepancy could be the definition of passive diastolic compliance used in the current study. While the ESPVR can be regarded as relatively linear, the EDPVR is known to be inherently nonlinear (Burkhoff et al., 2005). In this context, the constant passive compliance described by the time-varying elastance implies a linear EDPVR, thereby leading to inaccuracies in the estimated value. In contrast, in the same study, Schmitt et al. (2009) showed no differences in diastolic compliance with dobutamine in patients with a single ventricle. The validity of our results should be assessed in future studies by comparison with invasive pressure measurements, possibly acquired in patients that undergo catheterization as part of the clinical routine.

### Limitations

The current study has several limitations besides the ones mentioned in our previous publication (Casas et al., 2017). A major limitation is the lack of invasive measurements for validation of the estimated model-based parameters. This would require acquiring a family of pressure-volume loops by the use of catheter measurements during the infusion of dobutamine simultaneous to the CMR scan and was considered beyond the scope of the study. A second limitation is related to the uncertainty in the input measurements to the model. Our approach relies on non-invasive measurements of blood pressure and assumes the absence of both noise in the 4D Flow MRI data and potential errors derived from the analysis (i.e., inter- and intra- observer variability). Further studies are needed to assess how these potential sources of error might affect the uncertainty of the model-derived parameters. Nevertheless, given the trends in the parameters and the sufficiently large differences between the parameter values at rest and under dobutamine stress, we consider that these potential errors do not affect the validity of our results.

The study only included a limited number of subjects. This limits the statistical power of the study and the extrapolation of our results. This should therefore be taken into consideration when interpreting the statistical significance of the results. Nevertheless, the study group displays a broad range of physiologically relevant loads and heart rates, and the changes in parameters were significant despite the low total study population. Additional studies including a larger number of subjects should be conducted to confirm the clinical relevance of the statistical significances reported in the current work.

In addition, we only evaluated the changes in parameters with stress in subjects without known cardiovascular disease. The analysis of these parameters in subjects with a spectrum of diseases, such as ischemic heart disease, valvular disorders, and heart failure is subject to further studies. Those studies should focus on assessing the added value of these parameters over conventional image-derived parameters in diagnosing and optimizing the treatment of the disease.

## Conclusions

The integrated imaging-modeling approach used in this study provided subject-specific parameter values that behaved appropriately, according to previously reported effects of dobutamine on cardiovascular function, across a physiologically relevant range of loads and heart rates. With further validation, the approach could be used to assess these parameters, which are difficult to obtain non-invasively in clinical practice, thereby complementing conventional non-invasive indices used in the assessment of ventricular function. Future validation studies should preferably involve comparison to invasive measurements and application on larger populations, including both healthy subjects, and patients representing a spectrum of cardiovascular diseases. The personalized assessment may have the potential to generate advanced metrics of cardiovascular physiology and pathophysiology that could extend beyond conventional techniques for both diagnosis and optimization of a personalized medical regimen.

## Data availability

The datasets for this manuscript are not publicly available due to restrictions in the ethical approval. Requests to access the datasets should be directed to the corresponding author.

## Author contributions

BC, AB, MK, C-JC, and TE participated in the conception and design of the study. C-JC performed the recruitment of patients and carried out the data acquisition. BC and FV were involved in the analysis of the data. BC, GC, and TE participated in the implementation of the method. BC performed the computational analysis and drafted the manuscript. BC, GC, AB, MK, C-JC, and TE interpreted the results. All authors edited and revised the manuscript. All authors read and approved the final version of the manuscript.

### Conflict of interest statement

The authors declare that the research was conducted in the absence of any commercial or financial relationships that could be construed as a potential conflict of interest.

## Abbreviations

bSSFP: Balanced Steady-State Free Precession
CMR: Cardiac Magnetic Resonance
DBP: Diastolic Blood Pressure
CO: Cardiac Output
EDPVR: End-Diastolic Pressure-Volume Relationship
EOA: Effective Orifice Area
ESPVR: End-Systolic Pressure-Volume Relationship
EDV: End-diastolic volume
EF: Ejection fraction
ESV: End-systolic volume
LV: Left Ventricular
MAP: Mean Arterial Pressure
NYHA: New York Heart Association
PC: Phase-Contrast
PP: Pulse Pressure
RMSE: Root mean square error
SBP: Systolic Blood Pressure
SENSE: Sensitivity Enconding
SV: Stroke Volume
SVR: Systemic Vascular Resistance
VENC: Velocity Encoding
VTI: Velocity-Time Integral.

## References

[B1] Barnett D. B. (1989). Myocardial beta-adrenoceptor function and regulation in heart failure: implications for therapy. Br. J. Clin. Pharmacol. 27, 527–537. 2547407 10.1111/j.1365-2125.1989.tb03414.x PMC1379917

[B2] Baumgartner H. Stefenelli T. Niederberger J. Schima H. Maurer G. (1999). “Overestimation” of catheter gradients by doppler ultrasound in patients with aortic stenosis: a predictable manifestation of pressure recovery. J. Am. Coll. Cardiol. 33, 1655–1661. 10.1016/S0735-1097(99)00066-2 10334438

[B3] Binkley P. F. Murray K. D. Watson K. M. Myerowitz P. D. Leier C. V. (1991). Dobutamine increases cardiac output of the total artificial heart. Implications for vascular contribution of inotropic agents to augmented ventricular function. Circulation 84, 1210–1215. 10.1161/01.cir.84.3.1210 1884450

[B4] Broomé M. Maksuti E. Bjällmark A. Frenckner B. Janerot-Sjöberg B. (2013). Closed-loop real-time simulation model of hemodynamics and oxygen transport in the cardiovascular system. Biomed. Eng. Online 12:69. 10.1186/1475-925X-12-69 23842033 PMC3751725

[B5] Burattini R. Natalucci S. (1998). Complex and frequency-dependent compliance of viscoelastic windkessel resolves contradictions in elastic windkessels. Med. Eng. Phys. 20, 502–514. 10.1016/S1350-4533(98)00055-1 9832026

[B6] Burkhoff D. Mirsky I. Suga H. (2005). Assessment of systolic and diastolic ventricular properties via pressure-volume analysis: a guide for clinical, translational, and basic researchers. Am. J. Physiol. Hear. Circ. Physiol. 289, H501–H512. 10.1152/ajpheart.00138.2005 16014610

[B7] Casas B. Lantz J. Viola F. Cedersund G. Bolger A. F. Carlhäll C.-J.. (2017). Bridging the gap between measurements and modelling: a cardiovascular functional avatar. Sci. Rep. 7:6214. 10.1038/s41598-017-06339-0 28740184 PMC5524911

[B8] Chabiniok R. Moireau P. Lesault P. F. Rahmouni A. Deux J. F. Chapelle D. (2012). Estimation of tissue contractility from cardiac cine-MRI using a biomechanical heart model. Biomech. Model. Mechanobiol. 11, 609–630. 10.1007/s10237-011-0337-8 21796413

[B9] Chung C. S. Karamanoglu M. Kovács S. J. (2004). Duration of diastole and its phases as a function of heart rate during supine bicycle exercise. Am. J. Physiol. Hear. Circ. Physiol. 287, H2003–H2008. 10.1152/ajpheart.00404.2004 15217800

[B10] Danilouchkine M. G. Westenberg J. J. de Roos A. Reiber J. H. Lelieveldt B. P. (2005). Operator induced variability in cardiovascular MR: left ventricular measurements and their reproducibility. J. Cardiovasc. Magn. Reson. 7, 447–457. 10.1081/JCMR-200053578 15881528

[B11] Dyverfeldt P. Bissell M. Barker A. J. Bolger A. F. Carlhäll C.-J. Ebbers T.. (2015). 4D flow cardiovascular magnetic resonance consensus statement. J. Cardiovasc. Magn. Reson. 17, 1–19. 10.1186/s12968-015-0174-5 26257141 PMC4530492

[B12] Dyverfeldt P. Kvitting J.-P. Sigfridsson A. Engvall J. Bolger A. F. Ebbers T. (2008). Assessment of fluctuating velocities in disturbed cardiovascular blood flow: *in vivo* feasibility of generalized phase-contrast MRI. J. Magn. Reson. Imaging 28, 655–663. 10.1002/jmri.21475 18777557

[B13] Ebbers T. Haraldsson H. Dyverfeldt P. Sigfridsson A. Warntjes M. J. B. Wigström L. (2008). Higher order weighted least-squares phase offset correction for improved accuracy in phase-contrast MRI, in Proceedings ISMRM (Toronto, ON).

[B14] Ellwein L. M. Tran H. T. Zapata C. Novak V. Olufsen M. S. (2008). Sensitivity analysis and model assessment: mathematical models for arterial blood flow and blood pressure. Cardiovasc. Eng. 8, 94–108. 10.1007/s10558-007-9047-3 18080757

[B15] el-Said E.-S. Roelandt J. R. Fioretti P. M. McNeill A. J. Forster T. Boersma H.. (1994). Abnormal left ventricular early diastolic filling during dobutamine stress Doppler echocardiography is a sensitive indicator of significant coronary artery disease. J. Am. Coll. Cardiol. 24, 1618–1624. 10.1016/0735-1097(94)90165-1 7963106

[B16] Eriksson J. Carlhäll C. J. Dyverfeldt P. Engvall J. Bolger A. F. Ebbers T. (2010). Semi-automatic quantification of 4D left ventricular blood flow. J. Cardiov. Magn. Reson. 12, 1–10. 10.1186/1532-429x-12-9 20152026 PMC2831022

[B17] Garcia D. Pibarot P. Durand L.-G. (2005). Analytical modeling of the instantaneous pressure gradient across the aortic valve. J. Biomech. 38, 1303–1311. 10.1016/j.jbiomech.2004.06.018 15863115

[B18] Garcia J. Marrufo O. R. Rodriguez A. O. Larose E. Pibarot P. Kadem L. (2012). Cardiovascular magnetic resonance evaluation of aortic stenosis severity using single plane measurement of effective orifice area. J. Cardiovasc. Magn. Reson. 14:23. 10.1186/1532-429X-14-23 22480269 PMC3366866

[B19] Gurev V. Lee T. Constantino J. Arevalo H. Trayanova N. A. (2011). Models of cardiac electromechanics based on individual hearts imaging data: image-based electromechanical models of the heart. Biomech. Model. Mechanobiol. 10, 295–306. 10.1007/s10237-010-0235-5 20589408 PMC3166526

[B20] Heiberg E. Sjögren J. Ugander M. Carlsson M. Engblom H. Arheden H. (2010). Design and validation of segment - freely available software for cardiovascular image analysis. BMC Med. Imaging 10:1. 10.1186/1471-2342-10-1 20064248 PMC2822815

[B21] Heldt T. Shim E. B. Kamm R. D. Mark R. G. (2002). Computational modeling of cardiovascular response to orthostatic stress. J. Appl. Physiol. 92, 1239–1254. 10.1152/japplphysiol.00241.2001 11842064

[B22] Iwakami M. Hashimoto Y. Oniki T. Numano F. (1996). Effects of dobutamine on left ventricular diastolic performance are attenuated in patients with systemic hypertension. Am. J. Cardiol. 78, 369–372. 10.1016/S0002-9149(96)00298-6 8759825

[B23] Karliner J. S. Lewinter M. M. Mahler F. Engler R. O'Rourke R. A. (1977). Pharmacologic and hemodynamic influences on the rate of isovolumic left ventricular relaxation in the normal conscious dog. J. Clin. Invest. 60, 511–521. 10.1172/JCI108803 893662 PMC372396

[B24] Kilner P. J. Henein M. Y. Gibson D. G. (1997). Our tortuous heart in dynamic mode — an echocardiographic study of mitral flow and movement in exercising subjects. Heart Vessels 12, 103–110. 10.1007/bf02767127 9496460

[B25] Krishnamurthy A. Villongco C. T. Chuang J. Frank L. R. Nigam V. Belezzuoli E.. (2013). Patient-specific models of cardiac biomechanics. J. Comput. Phys. 244, 4–21. 10.1016/j.jcp.2012.09.015 23729839 PMC3667962

[B26] Lamata P. Casero R. Carapella V. Niederer S. A. Bishop M. J. Schneider J. E.. (2014). Images as drivers of progress in cardiac computational modelling. Prog. Biophys. Mol. Biol. 115, 198–212. 10.1016/j.pbiomolbio.2014.08.005 25117497 PMC4210662

[B27] Liang C. S. Hood W. B. (1979). Dobutamine infusion in conscious dogs with and without autonomic nervous system inhibition: effects on systemic hemodynamics, regional blood flows and cardiac metabolism. J. Pharmacol. Exp. Ther. 211, 698–705. 512933

[B28] Little W. C. Rassi A. Jr Freeman G. L. (1987). Comparison of effects of dobutamine and ouabain on left ventricular contraction and relaxation in closed-chest dogs. J. Clin. Invest. 80, 613–620. 10.1172/JCI113113 3624480 PMC442282

[B29] Maceira A. M. Prasad S. K. Khan M. Pennell D. J. (2006). Normalized left ventricular systolic and diastolic function by steady state free precession cardiovascular magnetic resonance. J. Cardiovasc. Magn. Reson. 8, 417–426. 10.1080/10976640600572889 16755827

[B30] Mancia G. Fagard R. Narkiewicz K. Redon J. Zanchetti A. Böhm M.. (2013). 2013 ESH/ESC Guidelines for the management of arterial hypertension the task force for the management of arterial hypertension of the European Society of Hypertension (ESH) and of the European Society of Cardiology (ESC). Eur. Heart J. 34, 2159–2219. 10.1093/eurheartj/eht151 23771844

[B31] Muthurangu V. Razavi R. S. (2005). The value of magnetic resonance guided cardiac catheterisation. Heart 91, 995–996. 10.1136/hrt.2004.055137 16020579 PMC1769020

[B32] Mynard J. P. Davidson M. R. Penny D. J. Smolich J. J. (2012). A simple, versatile valve model for use in lumped parameter and one-dimensional cardiovascular models. Int. J. Numer. Method Biomed. Eng. 28, 626–641. 10.1002/cnm.1466 25364842

[B33] Myreng Y. Smiseth O. A. (1990). Assessment of left ventricular relaxation by Doppler echocardiography. Comparison of isovolumic relaxation time and transmitral flow velocities with time constant of isovolumic relaxation. Circulation 81, 260–266. 10.1161/01.cir.81.1.260 2297830

[B34] Otsuki T. Maeda S. Iemitsu M. Saito Y. Tanimura Y. Ajisaka R.. (2008). Systemic arterial compliance, systemic vascular resistance, and effective arterial elastance during exercise in endurance-trained men. Am. J. Physiol. Regul. Integr. Comp. Physiol. 295, R228–R235. 10.1152/ajpregu.00009.2008 18463196

[B35] Paelinck B. P. Lamb H. J. Bax J. J. van der Wall E. E. de Roos A. (2004). MR flow mapping of dobutamine-induced changes in diastolic heart function. J. Magn. Reson. Imaging 19, 176–181. 10.1002/jmri.10448 14745750

[B36] Parker J. D. Landzberg J. S. Bittl J. A. Mirsky I. Colucci W. S. (1991). Effects of beta-adrenergic stimulation with dobutamine on isovolumic relaxation in the normal and failing human left ventricle. Circulation 84, 1040–1048. 10.1161/01.cir.84.3.1040 1653121

[B37] Pennell D. J. Underwood S. R. Manzara C. C. Swanton R. H. Walker J. M. Ell P. J.. (1992). Magnetic resonance imaging during dobutamine stress in coronary artery disease. Am. J. Cardiol. 70, 34–40. 10.1016/0002-9149(92)91386-i 1615867

[B38] Roest A. A. Kunz P. Lamb H. J. Helbing W. A. van der Wall E. E. De Roos A. (2001). Biventricular response to supine physical exercise in young adults assessed with ultrafast magnetic resonance imaging. Am. J. Cardiol. 87, 601–605. 10.1016/s0002-9149(00)01438-7 11230846

[B39] Schmitt B. Steendijk P. Lunze K. Ovroutski S. Falkenberg J. Rahmanzadeh P.. (2009). Integrated assessment of diastolic and systolic ventricular function using diagnostic cardiac magnetic resonance catheterization. JACC Cardiovasc. Imaging 2, 1271–1281. 10.1016/j.jcmg.2009.09.007 19909930

[B40] Senzaki H. Chen C. H. Kass D. A. (1996). Single-beat estimation of end-systolic pressure-volume relation in humans. A new method with the potential for noninvasive application. Circulation 94, 2497–2506. 10.1161/01.cir.94.10.2497 8921794

[B41] Sievers B. Kirchberg S. Bakan A. Franken U. Trappe H. (2004). Impact of papillary muscles in ventricular volume and ejection fraction assessment by cardiovascular magnetic resonance. J. Cardiovasc. Magn. Reson. 6, 9–16. 10.1081/JCMR-120027800 15054924

[B42] Smith N. de Vecchi A. McCormick M. Nordsletten D. Camara O. Frangi A. F.. (2011). euHeart: personalized and integrated cardiac care using patient-specific cardiovascular modelling. Interface Focus 1, 349–364. 10.1098/rsfs.2010.0048 22670205 PMC3262448

[B43] Stalder A. F. Russe M. F. Frydrychowicz A. Bock J. Hennig J. Markl M. (2008). Quantitative 2D and 3D phase contrast MRI: optimized analysis of blood flow and vessel wall parameters. Magn. Reson. Med. 60, 1218–1231. 10.1002/mrm.21778 18956416

[B44] Steding-Ehrenborg K. Jablonowski R. Arvidsson P. M. Carlsson M. Saltin B. Arheden H. (2013). Moderate intensity supine exercise causes decreased cardiac volumes and increased outer volume variations: a cardiovascular magnetic resonance study. J. Cardiovasc. Magn. Reson. 15:96. 10.1186/1532-429x-15-96 24156367 PMC4015552

[B45] Stergiopulos N. Meister J. J. Westerhof N. (1996). Determinants of stroke volume and systolic and diastolic aortic pressure. Am. J. PHysiol. Heart C. 270, H2050–H2059. 8764256 10.1152/ajpheart.1996.270.6.H2050

[B46] Suga H. Sagawa K. Shoukas A. A. (1973). Load independence of the instantaneous pressure-volume ratio of the canine left ventricle and effects of epinephrine and heart rate on the ratio. Circ. Res. 32, 314–322. 10.1161/01.res.32.3.314 4691336

[B47] Sun Y. Sjöberg B. J. Ask P. Loyd D. Wranne B. (1995). Mathematical model that characterizes transmitral and pulmonary venous flow velocity patterns. Am. J. Physiol. 268, H476–H489. 7840296 10.1152/ajpheart.1995.268.1.H476

[B48] Thomas J. D. Popović Z. B. (2006). Assessment of left ventricular function by cardiac ultrasound. J. Am. Coll. Cardiol. 48, 2012–2025. 10.1016/j.jacc.2006.06.071 17112991

[B49] Trayanova N. A. Constantino J. Gurev V. (2011). Electromechanical models of the ventricles. Am. J. Physiol. Hear. Circ. Physiol. 301, H279–H286. 10.1152/ajpheart.00324.2011. 21572017 PMC3154669

[B50] Tuttle R. R. Mills J. (1975). Dobutamine: development of a new catecholamine to selectively increase cardiac contractility. Circ. Res. 36, 185–196. 10.1161/01.res.36.1.185 234805

[B51] Udelson J. E. Bacharach S. L. Cannon R. O. Bonow R. O. (1990). Minimum left ventricular pressure during beta-adrenergic stimulation in human subjects. Evidence for elastic recoil and diastolic “suction” in the normal heart. Circulation 82, 1174–1182. 10.1161/01.cir.82.4.1174 1976048

[B52] van Rugge F. P. van der Wall E. E. Spanjersberg S. J. de Roos A. Matheijssen N. A. Zwinderman A. H.. (1994). Magnetic resonance imaging during dobutamine stress for detection and localization of coronary artery disease. Quantitative wall motion analysis using a modification of the centerline method. Circulation 90, 127–138. 10.1161/01.cir.90.1.127 8025988

[B53] Vandervoort P. M. Greenberg N. L. Powell K. A. Cosgrove D. M. Thomas J. D. (1995). Pressure recovery in bileaflet heart valve prostheses: localized high velocities and gradients in central and side orifices with implications for doppler-catheter gradient relation in aortic and mitral position. Circulation 92, 3464–3472. 10.1161/01.cir.92.12.3464 8521568

[B54] Westenberg J. J. Roes S. D. Ajmone Marsan N. Binnendijk N. M. Doornbos J. Bax J. J.. (2008). Mitral valve and tricuspid valve blood flow: accurate quantification with 3D velocity-encoded MR imaging with retrospective valve tracking. Radiology 249, 792–800. 10.1148/radiol.2492080146 18849503

[B55] Westermann D. Kasner M. Steendijk P. Spillmann F. Riad A. Weitmann K.. (2008). Role of left ventricular stiffness in heart failure with normal ejection fraction. Circulation 117, 2051–2060. 10.1161/circulationaha.107.716886 18413502

[B56] Xiang Q.-S. (1995). Temporal phase unwrapping for cine velocity imaging. J. Magn. Reson. Imaging 5, 529–534. 10.1002/jmri.1880050509 8574036

[B57] Zhong L. Ghista D. N. Ng E. Y. Lim S. T. (2005). Passive and active ventricular elastances of the left ventricle. Biomed. Eng. Online 4:10. 10.1186/1475-925X-4-10 15707494 PMC549522

[B58] Zile M. R. Baicu C. F. Gaasch W. H. (2004). Diastolic heart failure — abnormalities in active relaxation and passive stiffness of the left ventricle. N. Engl. J. Med. 350, 1953–1959. 10.1056/NEJMoa032566 15128895

