# Supplementary material for: Non-invasive Assessment of Systolic and Diastolic Cardiac Function During Rest and Stress Conditions Using an Integrated Image-Modeling Approach
Source: Front Physiol. 2018 Oct 30;9:1515. doi: 10.3389/fphys.2018.01515 (PMC6218619; doi:10.3389/fphys.2018.01515)
Supplement: Supplementary file 1 [file Table_1.pdf]

# *Supplementary Material*

# Non-invasive assessment of systolic and diastolic left ventricular function during rest and stress conditions using an integrated image-modelling approach

Belén Casas[1,2], Federica Viola[1], Gunnar Cedersund[4], Ann F. Bolger[1,5], Matts Karlsson[2,6], Carl-Johan Carlhäll[1,2,3], Tino Ebbers[1,2]*

1 - Division of Cardiovascular Medicine, Department of Medical and Health Sciences, Linköping University, Linköping, Sweden.
2 - Center for Medical Image Science and Visualization (CMIV), Linköping University, Linköping, Sweden.
3 - Department of Clinical Physiology, Department of Medical and Health Sciences, Linköping University, Linköping, Sweden.
4 - Department of Biomedical Engineering, Linköping University, Linköping, Sweden.
5 - Department of Medicine, University of California San Francisco, San Francisco, California, USA.
6 - Division of Applied Thermodynamics and Fluid Mechanics, Department of Management and Engineering, Linköping University, Linköping, Sweden

* **Correspondence:** Tino Ebbers: tino.ebbers@liu.se

## 1 Methods

### 1.1 Model parameters

**Table S1:** Parameters in the lumped-parameter model, including parameter values defined in literature.

| Parameter | Description (units) | Literature values |
|---|---|---|
| **Pulmonary venous system** | | |
| $P_{pu}$ | Pulmonary capillary pressure (mmHg) | 7.4 (Sun et al.) |
| $R_{pv}$ [a] | Resistance of pulmonary veins (mmHg·s/mL) | $2\cdot10^{-3}$ (Sun et al.) |
| $L_{pv}$ [a] | Inertance of pulmonary veins (mmHg· s$^2$/mL) | $5\cdot10^{-4}$ (Sun et al.[14]) |
| $R_{pvc}$ [a] | Viscoelastic resistance of pulmonary capillaries and veins (mmHg·s/mL) | 0.01 (Sun et al.[14]) |
| $C_{pvc}$ [a] | Capacitance of pulmonary capillaries and veins (mL/mmHg) | 4 (Sun et al.[14]) |

| | | |
|---|---|---|
| $R_{pu}$ [a] | Resistance of pulmonary capillaries (mmHg·s / mL) | 0.01 (Sun et al.[14]) |
| **Heart parameters** | | |
| **Left atrium (LA)** | | |
| $K_{s,LA}$ | Source resistance coefficient of the LA (s/mL) | $10 \cdot 10^{-9}$ (Mynard et al.[15]) |
| $E_{min,LA}$ | Minimal elastance of the LA (mmHg/mL) | 0.08 (Mynard et al.[15]) |
| $E_{max,LA}$ | Maximal elastance of the LA (mmHg/mL) | 0.17 (Mynard et al.[15]) |
| $V_{0,LA}$ [a] | Unstressed volume of the LA (mL) | 3 (Mynard et al.[15]) |
| $R_{C,LA}$ | Contraction rate constant of the LA (-) | 1.32 (Mynard et al.[15]) |
| $R_{R,LA}$ | Relaxation rate constant of the LA (-) | 13.1 (Mynard et al.[15]) |
| $\alpha_{1,LA}$ | Systolic time constant shape factor of the LA (-) | 0.11 (Mynard et al.[15]) |
| $\alpha_{2,LA}$ | Diastolic time constant shape factor of the LA (-) | 0.18 (Mynard et al.[15]) |
| $R_{visc,LA}$ [a] | Viscous loss resistance for the LA (mmHg·s / mL) | $1 \cdot 10^{-4}$ (Mynard et al.[15]) |
| $Onset_{LA}$ | Onset of contraction of the LA (s) | 0.85 (Mynard et al.[15]) |
| **Left ventricle (LV)** | | |
| $K_{s,LV}$ [a] | Source resistance coefficient of the LV (s/mL) | $4 \cdot 10^{-9}$ (Mynard et al.[15]) |
| $E_{min,LV}$ | Minimal elastance of the LV (mmHg/mL) | 0.08 (Mynard et al.[15]) |
| $E_{max,LV}$ | Maximal elastance of the LV (mmHg/mL) | 3 (Mynard et al.[15]) |
| $V_{0,LV}$ [a] | Unstressed volume of the LV (mL) | 10 (Mynard et al.[15]) |
| $R_{C,LV}$ | Contraction rate constant of the LV (-) | 1.32 (Mynard et al.[15]) |
| $R_{R,LV}$ | Relaxation rate constant of the LV (-) | 27.4 (Mynard et al.[15]) |
| $\alpha_{1,LV}$ | Systolic time constant shape factor of the LV (-) | 0.269 (Mynard et al.[15]) |

| | | |
|---|---|---|
| $\alpha_{2,LV}$ | Diastolic time constant shape factor of the LV (-) | 0.452 (Mynard et al.[15]) |
| $R_{visc,LV}$[b] | Viscous loss resistance for the LV (mmHg·s/mL) | $1 \cdot 10^{-4}$ (Mynard et al.[15]) |
| $Onset_{LV}$ | Onset of contraction of the LV (s) | 0 (Mynard et al.[15]) |
| **Mitral valve** | | |
| $R_{mv}$ | Resistance of the mitral valve (mmHg·s/mL) | $3.75 \cdot 10^{-3}$ (Sun et al.[14]) |
| $L_{mv}$ | Inertance of the mitral valve (mmHg·s$^2$/mL) | $2 \cdot 10^{-4}$ (Sun et al.[14]) |
| **Aortic valve** | | |
| $EOA_{av}$ | Effective orifice area of the aortic valve (cm$^2$) | 1.69 (Garcia et al.[16]) |
| $A_{ao}$ | Cross sectional area of the aorta (cm$^2$) | 5 (Olufsen et al.[64]) |
| $L_{av}$ | Inertance of the aortic valve (mmHg·s$^2$/mL) | $4 \cdot 10^{-4}$ (Sun et al.[14]) |
| **Systemic arterial system** | | |
| $R_{aa}$ | Resistance of the ascending aorta (mmHg·s/mL) | 0.04 (Sun et al.[14]), |
| $L_{aa}$ | Inertance of the ascending aorta (mmHg· s$^2$/mL) | $5 \cdot 10^{-4}$ (Sun et al.[14]), |
| $R_{aav}$ | Viscoelastic resistance for $C_{aa}$ (mmHg·s/mL) | 0.01 (Sun et al.[14]) |
| $C_{aa}$ | Capacitance of the ascending aorta (mL/mmHg) | 0.1 (Sun et al.[14]) |
| $R_{pc}$ | Viscoelastic resistance for $C_{pc}$ (mmHg·s/mL) | - |
| $R_{pr}$ | Peripheral resistance (mmHg·s/mL) | 1.2 (Sun et al.[14]), |
| $C_{pc}$ | Peripheral compliance (mL/mmHg) | 2 (Sun et al.[14]) |
| **Other parameters** | | |
| $T$[b] | Duration of the cardiac cycle (s) | - |
| $\rho$ | Density of blood (g/mL) | 1.06 |

## 2 Results

**Table S2:** Subject-specific fitting results for the nine subjects included in the study. The agreement between the measured and the model-generated waveforms is assessed using the root mean square error (RMSE). MV: mitral valve; AV: aortic valve; AA: ascending aorta.

| | **RMSE (ml/s)** | | | | | |
|---|---|---|---|---|---|---|
| | **Rest** | | | **Dobutamine** | | |
| **Subject** | **MV** | **AV** | **AA** | **MV** | **AV** | **AA** |
| 1 | 29.7 | 25.8 | 21.5 | 58.3 | 66.8 | 31.1 |
| 2 | 32.4 | 26.1 | 23.0 | 58.0 | 29.5 | 36.2 |
| 3 | 22.7 | 16.1 | 23.3 | 49.4 | 51.9 | 35.6 |
| 4 | 27.7 | 17.2 | 20.6 | 77.6 | 29.0 | 21.5 |
| 5 | 15.4 | 19.6 | 18.7 | 32.3 | 35.6 | 25.1 |
| 6 | 34.8 | 30.4 | 33.1 | 76.6 | 82.4 | 47.9 |
| 7 | 24.3 | 19.7 | 24.9 | 44.1 | 82.7 | 46.1 |
| 8 | 18.4 | 16.0 | 16.0 | 60.5 | 46.1 | 62.2 |
| 9 | 20.0 | 18.6 | 19.0 | 22.1 | 24.8 | 27.5 |

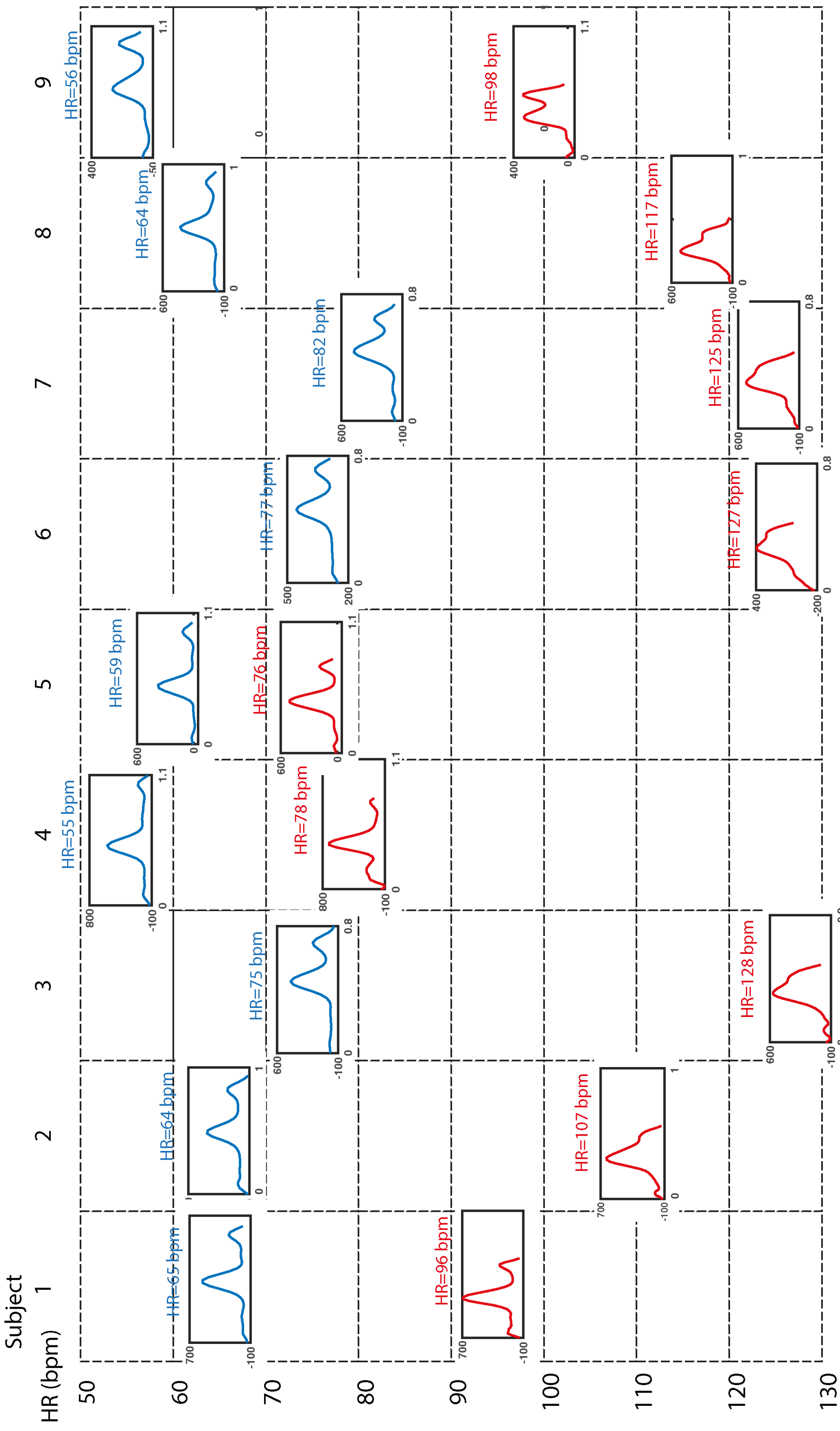


**Figure S1:** Changes in 4D Flow MRI-derived mitral flow waveforms with increasing heart rates for all study subjects. For each subject, the mitral flow patterns at rest (blue) and after dobutamine infusion (red) are included. The x and y-axis of the volumetric flow waveforms indicate time (s) and flow (ml/s), respectively.
